# Understanding the mechanisms generating outcomes in a Danish peer support intervention for socially vulnerable people with type 2-diabetes: a realist evaluation

**DOI:** 10.1186/s13690-021-00676-3

**Published:** 2021-09-06

**Authors:** Stine Dandanell Garn, Charlotte Glümer, Sarah Fredsted Villadsen, Gritt Marie Hviid Malling, Ulla Christensen

**Affiliations:** 1 Center for Diabetes, City of Copenhagen, Copenhagen, Denmark; 2 Department of Public Health, Section for Social Medicine, University of Copenhagen, Copenhagen, Denmark; 3 Public Health and Epidemiology Group, Department of Health Science and Technology, Aalborg University, Aalborg, Denmark

**Keywords:** Complex Intervention, Realist evaluation, Mechanisms, Context, Peer support, Diabetes self-management, Healthcare services, Inequality

## Abstract

**Background:**

Despite an increasing use and positive effects of peer support interventions, little is known about how the outcomes are produced. Thus, it is essential not only to measure outcomes, but also to identify the mechanisms by which they are generated. Using a realist evaluation approach, we aimed to identify the mechanisms generating outcomes in a Danish peer support intervention for socially vulnerable people with type 2-diabetes (peers). By investigating the participating peers’ interactions, we furthermore examined how their individual contextual factors either facilitated or hindered the mechanisms in operation.

**Methods:**

We used a multi-method case-study design (*n* = 9). Data included semi-structured interviews with four key groups of informants (peer, peer supporter, project manager, and a diabetes nurse) for each case (*n* = 25). Furthermore, we collected survey data from peers both before and after participation (*n* = 9). The interview data were analysed using a systematic text condensation, and the Intervention-context-actor-mechanism-outcome framework was used to structure the analysis.

**Results:**

We identified 2 groups of mechanisms that improved diabetes self-management and the use of healthcare services (outcomes): ‘perceived needs and readiness’ and ‘encouragement and energy’. However, the mechanisms only generated the intended outcomes among peers with a stable occupation and financial situation, a relatively good health condition, and sufficient energy (all defined as contextual factors). Independent of these contextual factors, ‘experience of social and emotional support’ was identified as a mechanism within all peers that increased self-care awareness (defined as output). Dependent on whether the contextual factors facilitated or hindered the mechanisms to generate outcomes, we categorised the peers into those who achieved outcomes and those who did not.

**Conclusions:**

We identified two groups of mechanisms that improved the peers’ diabetes self-management and use of healthcare services. The mechanisms only generated the intended outcomes if peers’ individual contextual factors facilitated an active interaction with the elements of the intervention. However, independent of these contextual factors, a third group of mechanisms increased self-care awareness among all peers. We highlight the importance of contextual awareness of the target groups in the design and evaluation of peer support interventions for socially vulnerable people with type 2-diabetes.

**Trial registration:**

ClinicalTrials.gov, Retrospective Registration (20 Jan 2021), registration number NCT04722289.

## Background

Peer support programmes are increasingly used worldwide as a supplement to the established healthcare system to support people with type 2-diabetes (T2D) in managing their disease [1–4]. Moreover, these are acknowledged by the World Health Organization as feasible and cost-effective interventions [5]. Peer support within the healthcare field refers to social, emotional, and practical assistance provided by non-professionals to help people adopt, change and maintain health behaviours [6, 7].

Several studies have found a positive effect of peer support programmes on diabetes self-management (DSM), such as meal planning, physical activity, taking medication, and blood glucose monitoring [8–11]. Furthermore, peer support has benefitted health outcomes, such as self-reported health status, emotional distress and glycaemic, and blood pressure control [8, 9, 11–13]. Finally, peer support is highlighted for its potential to support socially vulnerable people in managing their T2D [14–16]. Often, this group is referred to as people with low socioeconomic status, such as low levels of income, education, employment, and social relations [14, 16–18]. This population group has a higher risk of developing complications of T2D [15, 19], often face multiple barriers to accessing healthcare services [14, 15, 20] and experience worse health outcomes [21]. However, even though socioeconomic differences exist in access to healthcare services, as well as the treatment and consequences of T2D [16, 19, 20, 22], no studies have, to our knowledge, focused on the implementation of peer support programmes for socially vulnerable people with T2D.

In general, little is known about how peer support interventions produce outcomes. Methodological guidance on complex interventions emphasises the importance of focusing on what is implemented, including the mechanisms that generate outcomes and the contexts that influence their implementation [23, 24]. Using the realist evaluation (RE) approach developed by Pawson & Tilley [25], it is possible to identify outcome-generating mechanisms in complex health interventions such as peer support programmes. This can be achieved by investigating how the actors involved interact in the intervention and how contextual factors influence this. The RE’s main principle is that all complex interventions have underlying theories, referred to as programme theories that describe the relationship between context, mechanisms and outcomes (CMO-configurations) in the intervention. By developing and empirically testing a programme theory, it is possible to identify the parts of the intervention that has had an effect, in different contextual settings for the targeted populations [25]. Also, the “Intervention-Context-Actor-Mechanism-Outcome” (ICAMO) framework [26] is increasingly applied as a supplement [26–28]. The ICAMO is a modified version of Pawson & Tilley’s CMO-configuration, and it includes two extra components into the configuration: “intervention” and “actors”. Thus, using the ICAMO gives an explicit focus on how the actors involved interact in the intervention.

### Study aim

By using the RE approach, including the ICAMO-framework, we aimed to identify the mechanisms generating outcomes in a Danish peer support intervention, aiming to improve diabetes self-management and the use of healthcare services among socially vulnerable people with type 2-diabetes (peers). Based on a multi-method approach including quantitative survey and qualitative interviews, we investigated how the peers interacted in the intervention. Thus, we were able to focus on how their individual contextual factors facilitated or hindered the operation of mechanisms in the intervention.

## Methods

### Setting: the Danish healthcare system

The Danish healthcare system is universal and almost free of charge for Danish citizens, as all healthcare services are financed by general taxes [29]. It operates across three political and administrative levels: the state, five regions and 98 municipalities. The state is responsible for initiating and coordinating overall national health policies and legislation on healthcare. The regions are responsible for the planning and execution of diabetes care within hospitals and general practice, and the municipalities are responsible for diabetes rehabilitation outside hospitals, health promotion and disease prevention [30, 31].

### ‘Together on Diabetes’ intervention

Together on Diabetes is an ongoing intervention, developed and implemented in 2017 by Copenhagen Municipality’s Centre for Diabetes (CFD) and The Danish Diabetes Association (DDA). The intervention is part of the Copenhagen city action plan for T2D [30], and, the public-private partnership ‘Cities Changing Diabetes Copenhagen’ (CCDC) [31, 32].

Through social, emotional and practical support provided by non-professional volunteers with T2D (“peer supporters”), the intervention aims to improve DSM and increase the use of healthcare services among socially vulnerable people with T2D (peers). The intervention consists of five components: recruitment of peers and peer supporters; training of peer supporters; matchmaking between peers and peer supporters; 6 months of fortnightly individual face-to-face meetings between peers and peer supporters; and ongoing supervision and network meetings for peer supporters. The peer support meeting contains three activities: ‘social and emotional support’; ‘assistance in daily management’; and ‘linkage to healthcare services’ [33].

Based on an initial quantitative analysis of people with T2D in disadvantaged areas of Copenhagen with high diabetes prevalence [22], the inclusion criteria to participate as a peer were defined as poorly regulated T2D, multi-morbidity, no employment, low/no education, no contact to the healthcare system and living alone with no/minimal social network. The exclusion criterion was poorly regulated mental disease.

The inclusion criteria to become a peer supporter were defined as well-regulated T2D, basic knowledge about T2D and the Danish healthcare system, good communication skills, empathy and an interest in supporting a socially vulnerable person with T2D. These criteria were based on existing knowledge about peer support programmes [6, 7, 34].

As part of the intervention’s development and implementation, CFD and DDA formulated an initial programme theory that included the intervention components and theoretical assumptions of intended outcomes. Furthermore, it included potential contextual factors at an organisational, interpersonal and individual level that could affect the implementation. At an organisational level was contextual factors such as resources, coordination and communication with internal and external stakeholders and timing of the intervention. At the interpersonal and the individual level were contextual factors such as the relationship and chemistry between peer and peer supporter, the peers and peer supporters’ sociodemographic characteristics, health condition, other life events and social relations. The initial programme theory did not include mechanisms; hence, the aim of this study was to identify and investigate mechanisms.

### Conceptual framework

**Fig. 1:**
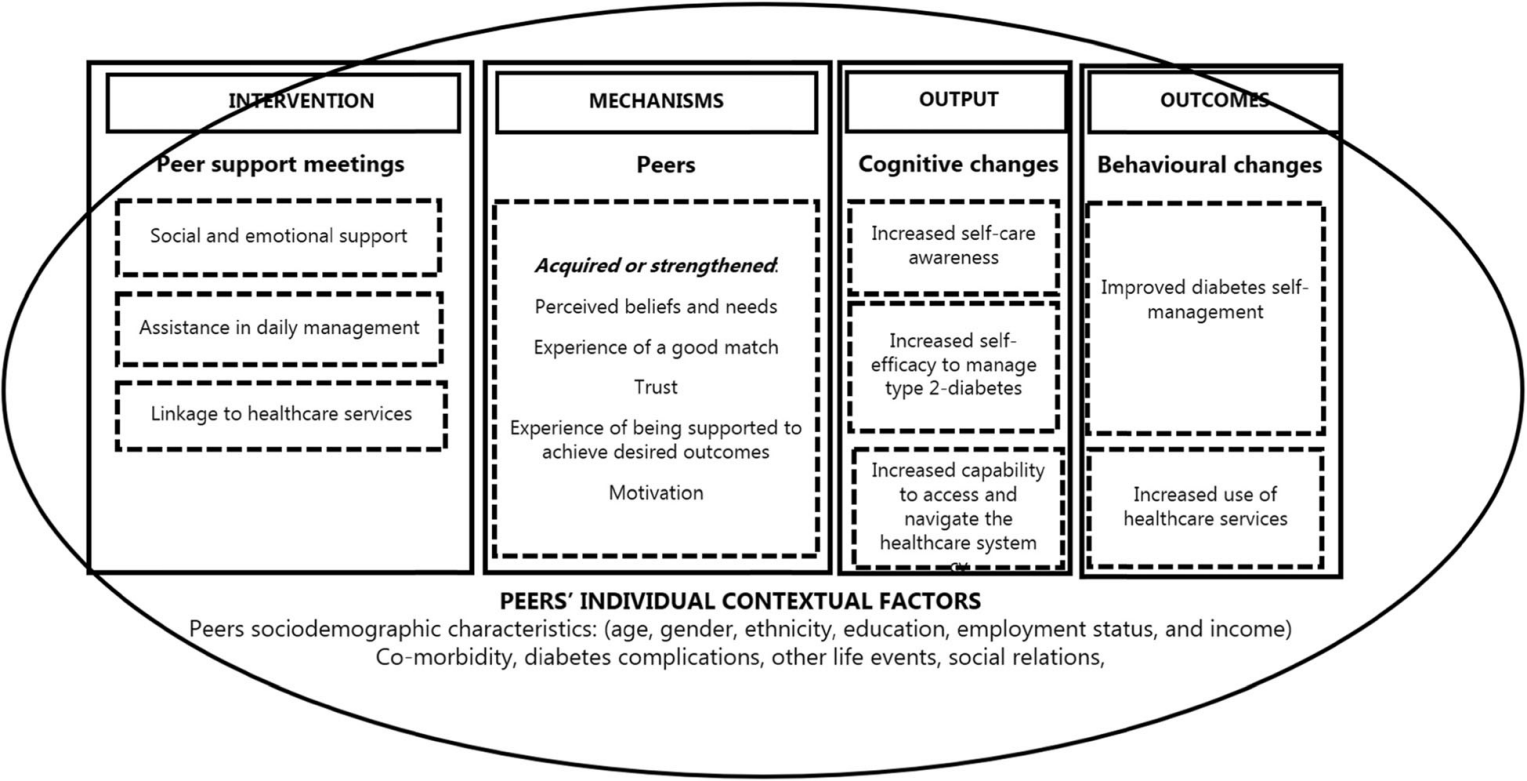
Initial ICAMO model

We chose to follow The Standards for Reporting Implementation Studies (StaRI) [35] in our presentation of our results. According to StaRI to understand and interpret effect, it is essential not only to measure the outcomes but also to underpin the mechanisms generating them and investigate the influence of the implementation context. Thus, we have focussed on the implementation process, based on the initial programme theory and relevant literature within the field [6, 7, 14, 16, 34]. We developed an initial ICAMO model for this study (Fig. 1) to structure our theoretical assumptions of how the intervention worked, for whom and under what conditions. The initial ICAMO model focused on a specific part of the intervention: the relationship between the three intervention activities in the peer support meetings, the mechanisms within peers that generated outcomes and the individual contextual factors in peers’ everyday lives that influenced how the mechanisms were at stake.

As illustrated in Fig. 1, we used Pawson’s definition of intermediate outputs (cognitive changes) and outcomes (behavioural changes) [36] to distinguish between whether the mechanisms generated both the intended cognitive and behavioural changes in terms of improved DSM and use of healthcare services, or if they solely generated cognitive changes. Moreover, we used Pawson’s four layers of contextual factors by Pawson [36] (the individual, interpersonal, organization and infrastructure layers), focusing on the individual layer, to investigate how the peers’ sociodemographic characteristics, capacities and life circumstances influenced how they interacted in the intervention. Finally, to define the term *‘mechanism’*, we applied the mechanism-framework by Dalkin et al. [37]. The framework is based on the original work of Pawson & Tilley [25] and defines mechanism as *“a combination of resources offered by the social programme under study and stakeholders’ reasoning in response”* [37]. Following this framework, a mechanism is both the resource that an intervention provides and the recipients’ reasoning and response to it. According to Dalkin et al. [37], this relationship between intervention resources that are introduced into a specific context enhances changes in the recipients’ reasoning that create the mechanisms that cause the outcomes, thereby making the intervention work.

### Study design, case selection and recruitment

We used a multi-method case study approach [38, 39] to identify the mechanisms that generated the intended outcomes. Furthermore, we used the ICAMO framework to structure our analysis.

In total 20 peers and 17 peer supporters participated in the Together on Diabetes intervention during the case study period (February 2018 to July 2019). All were invited by the project manager to participate in the study. 9 out of 12 pairs who completed the intervention during this period accepted the invitation and were thereby consequently selected as cases.

The majority of peers were recruited through CFD (*N* = 7). They were recruited among people who were considered too vulnerable to participate in CFD’s regular diabetes education and rehabilitation services. The remaining peers were recruited either by their general practitioner (GP) (*N* = 1) or through home care (*N* = 2). The peer supporters were recruited among people who had previously participated in CFD’s services (*N* = 5) by members from the DDA (*N* = 2), the GP (*N* = 1), or by people who applied via the ‘Together on Diabetes’ webpage (*N* = 2).

### Data collection

The study consisted of a quantitative survey and qualitative semi-structured interviews. Both were conducted between February 2018 and April 2020.

#### Surveys

A quantitative survey was conducted among peers at baseline and follow-up (*N* = 9) to measure both improvements in their DSM and use of healthcare services (outcomes).

Questions from the Danish National Health Survey [40] were used to measure the following indicators of DSM: Eating habits (consumption of vegetables, fruit, nuts, fish, and sugary and energy-dense food and beverages); Physical activity (time spent on different types of physical activities); Medication adherence and blood sugar monitoring. Furthermore, the use of healthcare services was measured by the number of times (during a 12-month period) the peers’ attended diabetes-related appointments with GPs, food therapists and ophthalmologists, or had other form of contact with relevant healthcare services. The survey data were used to inform the qualitative interviews on any improvements in these outcomes. Thus, to analyse and group the outcome-generating mechanisms emerged from the interviews. Furthermore, the survey data were used to obtain information on the peers’ individual contextual factors, such as their sociodemographic characteristics, co-morbidity, diabetes complications, social relation, and other life events. A diabetes nurse from CFD conducted the survey as a structured interview, as the peers were considered too vulnerable to complete the survey on their own. The diabetes nurse visited the peers in their own homes, both before and after the intervention. The peers received a handout version of the survey to be able to read the questions themselves.

#### Individual semi-structured interviews

We conducted 25 individual semi-structured interviews across the nine cases. The informants consisted of the peers (*n* = 9), peer supporters (*n* = 10), project manager and diabetes nurse. We interviewed each type of informant per case to obtain different perspectives on how the peers’ interacted in and benefited from the intervention. The interviews were conducted both immediately after the 6-month intervention and after the follow-up survey was completed.

The interview guide was semi-structured, based on survey data and the initial programme theory. The interviews consisted of questions about the following topics: peers and peer supporters’ reasons for participation; how they had been recruited; peers’ perceived needs towards the intervention; experiences of and activities conducted in the meetings; how they experienced peers’ cognitive (output) and behavioural changes (outcomes) as a result of the intervention; and whether any barriers or facilitators in peers’ contexts had affected their interaction in the intervention. In addition, the interviews with the project manager and diabetes nurse included questions about their reflections on patterns in outputs, outcomes and contextual factors across the nine cases. During the interviews, only the informant and interviewer were present. Interviews with peers took place in their own homes. Interviews with the other informants took place at CFD. However, due to the COVID-19 pandemic, seven interviews (peers (*n* = 3), peer supporters (*n* = 3) and project manager (*n* = 1)) were collected by telephone.

### Ethical considerations

The present study is part of a larger evaluation study of three diabetes interventions developed within the CCDC partnership programme [32]. The study was approved by the Danish Data Protection Agency (Rec. No: 2015-55-0630) and followed the codes of ethics in the Helsinki II Declaration. An ethical application was sent to The Research Ethics Committee for SCIENCE and HEALTH, at the Capital Region of Denmark. The Research Ethics Committee reported that they did not identify any ethical hindrances in conducting the study (Id. No: 18,029,206) and decided that no formal ethical approval was needed. All participants received written and verbal information about the study and gave written consent to participate. They were guaranteed anonymity and informed that they could withdraw from the study at any time, should they wish to do so.

### Data analysis

The semi-structured interviews were audio-recorded and transcribed verbatim. All transcripts were managed in NVivo 12 [41]. The Systematic Text Condensation [42] was used to analyse data and the ICAMO framework was used to structure the analytical findings [26].

The analysis consisted of four steps:

- Reading all transcripts to obtain an overall impression of data and identify preliminary themes related to the study aim.
- Identifying meaning units related to the preliminary themes. The first and last author then developed a set of codes, which they compared and discussed to clarify the relationship between the intervention activities, the mechanisms within peers that generated outcomes and the contextual factors in peers’ everyday lives that influenced how the mechanisms were at stake. This ensured confidence in the findings and facilitated agreement on an initial coding framework.
- Having coded all of the transcripts, we organised and synthesised data into themes with similar code groups. We then selected quotes to illustrate the findings.
- We revised our initial ICAMO model based on our empirical findings.

Throughout our study design, data collection and analysis, we followed the reporting standards for realist evaluations developed by the RAMESES II [43].

## Results

### Study participants

As illustrated in Table 1, the 9 peers included 7 males and 2 females. They were primarily male, middle-aged, of Danish origin, outside of the labour market, and with short and intermediate education backgrounds as well as multiple diagnoses and diabetes complications.

**Table 1:** Peers’ sociodemographic characteristics (Baseline survey data)

	Peers
**N**	9
**Sex**	
Male	7
Female	2
**Age**	
Below 50	1
50-65	6
Above 65	2
**Country of Birth:**	
Denmark	7
Other Western countries	1
Non-Western countries	1
**Source of income**	
Social security	4
State pension	2
Disability pension	3
**Education**	
Primary	4
Secondary	4
Higher	1
**Living situation**	
Alone	7
With children	1
With others	1
**Other diagnoses (N)**	
Mental health disorders	6
Arthritis	4
Oral health problems	3
KOL	2
Other chronic diagnoses	3
**Diabetes complications (N)**	
Cardiovascular diseases	7
Hypertension	5
Neuropati	4
Nefropati	4

The 10 peer supporters included 6 males and 4 females. They had different educational backgrounds, employment status and experience of working as a support volunteer. The diabetes nurse had 20 years of experience as a nurse and had previously worked as a home nurse. The project manager had 10 years of experience working with socially vulnerable groups and in peer support programmes.

### Differences in peers’ outcomes

**Fig. 2:**
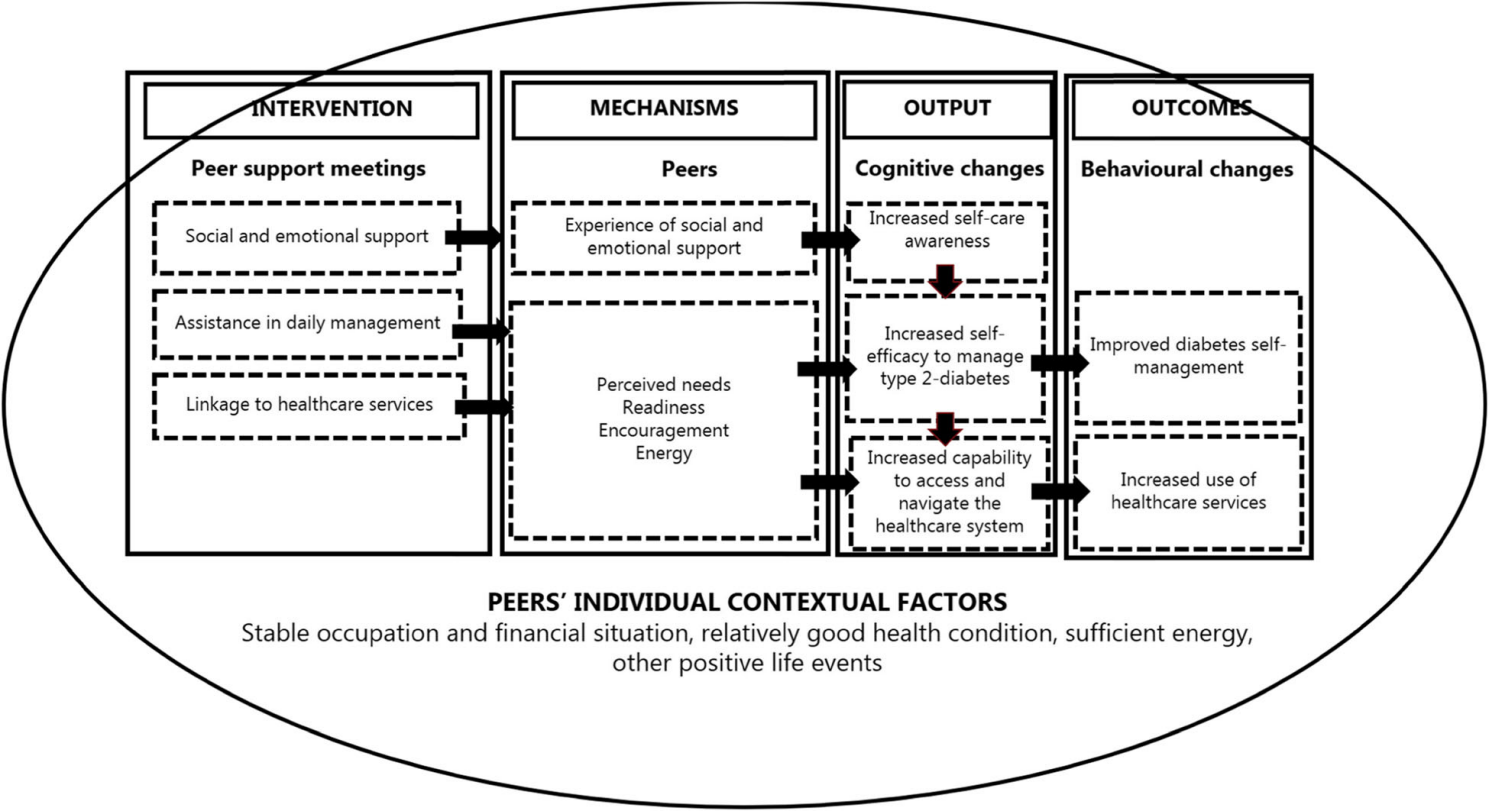
ICAMO model for peers who achieved the intended outcomes

**Fig. 3:**
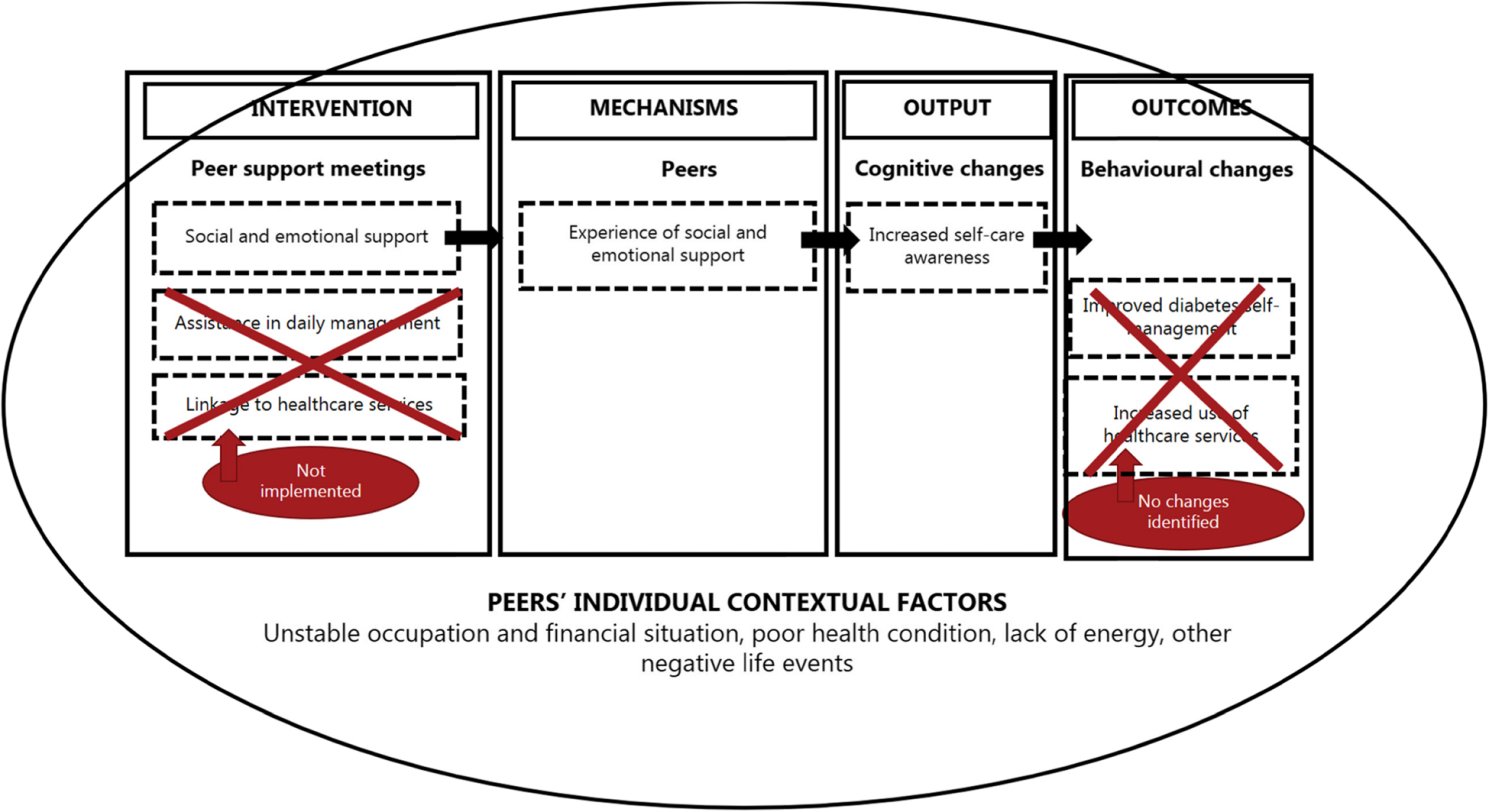
ICAMO model for peers who did not achieve the intended outcomes

Survey and interview data revealed large differences in the peers’ outcomes from the intervention. The intended outcomes (improved DSM and use of healthcare services) were only identified among four peers (See Figs. 2 and 3). A common pattern for those who achieved outcomes, compared to those who did not achieve any, was the implementation of a minimum of two of the three intervention activities in the peer support meetings. In the four cases, the peer supporters had been providing social and emotional support assisted with daily tasks (grocery shopping, cleaning, cooking healthy meals and exercising) and/or acted as a link to healthcare services (that is, being an observer at GP appointments and assisting in communication with other relevant healthcare services). Opposite, in the five cases in which none of intended outcomes where achieved, the peer support meetings had mainly consisted of social and emotional support.

### Contextual factors influencing variation in mechanisms and outcomes

Four main contextual factors in peers’ everyday lives were found to explain why some peers achieved the intended outcomes and others did not: ‘occupation and financial situation’, ‘health condition’, ‘energy’ and ‘other life events’) (See Figs. 2 and 3). The peers who achieved the intended outcomes were characterised by a stable occupation and financial situation, receiving a state or disability pension, combined with being in a better state of health. Moreover, some peers’ participation in other social activities during the intervention might have facilitated the outcomes. Conversely, for the peers who did not achieve any of the intended outcomes, these contextual factors functioned as barriers to how the peers interacted in the intervention. This group was characterised by an unstable occupation and financial situation, receiving social security benefits, combined with being in a worse state of health with severe pain due to multiple diabetes complications and both psychical and chronic mental diagnoses. In addition, some experienced negative life events during the intervention, such as accidental falls and the death or illness of close relatives, which interrupted their participation. Due to these barriers, which were on the individual contextual level, many peers described how they lacked energy to interact in the intervention.

### Mechanisms generating outputs and outcomes

Interview data revealed two groups of mechanisms within the peers that generated the intended outcomes: ‘perceived needs and readiness’ and ‘encouragement and energy’ (See Figs. 2 and 3). However, data showed a large variation in how these mechanisms operated depending on whether the contextual factors functioned as facilitators or barriers to the peers’ interactions in the intervention. Independent of the influence from the contextual factors, a third mechanism, ‘experience of social and emotional support’, was identified within all peers that increased self-care awareness (output).

#### Perceived needs and readiness

Peers’ perceived needs and readiness to interact in the intervention were found as interrelated mechanisms that generated both the intended cognitive (outputs) and behavioural changes (outcomes) in DSM and the use of healthcare services. However, as mentioned, these mechanisms operated differently between the peers depending on their context. For the peers where contextual factors in their everyday lives functioned as facilitators, a common pattern was that they had a perceived need for support in accessing and navigating the healthcare system and/or to improve central tasks in their DSM, for example, to get started with daily walks or cooking daily meals. In addition, they demonstrated a sufficient amount of readiness to interact in the intervention to meet these needs. This is illustrated in the following quote from one peer:

*“I needed a little help to get started cooking for myself because it had come to a complete standstill (…) To get started with grocery shopping so I could start making some proper food.“* (Peer, outcomes achieved).

In contrast, the peers’ perceived needs and readiness came into play more differently among those who were challenged by an unstable occupation and financial situation, a poor health condition, lack of energy and other negative life events. In general, they were less reflective about what they wanted to achieve with their participation. In the interviews, the majority expressed not having specific needs other than a need for social contact in their lives. Some mentioned not wanting to make health behaviour changes to improve their DSM or receive support in navigating the healthcare services. This is illustrated in the following case, where a peer explained how he did not want to stop drinking alcohol as he felt it did not matter anyway:

*“I don’t want to do that [stop drinking alcohol red.] (…) because I feel better when I drink. I am happy (…) Diabetes you have for life, no matter what you do, you know? So, so what? I am dying anyway.”* (Peer, no outcomes achieved).

According to the informants in this case, the peer’s poor life circumstances were caused by this lack of need and readiness to stop drinking alcohol. When entering the intervention, he had severe mental and physical challenges due to several chronic diagnoses and diabetes complications. Moreover, he had recently lost his job and had to focus his sparse energy on attending meetings at the jobcentre. In the interview, he mentioned that he was worried about being evicted from his home if he could not pay the next month’s rent. He had previously lived on the streets but was no longer capable of this due to his poor health condition. Thus, he was satisfied as long as he had a roof over his head.

*“Nothing is clear, you know? (…) They can send me a letter tomorrow stating that I will no longer receive cash benefits.”* (Peer, no outcomes achieved).

Because of the peers’ lack of need and readiness to achieve the intended outcomes, the peer support meetings mainly consisted of social and emotional support. This was a source of great frustration for many of the peer supporters, who felt unsuccessful in their roles while not being able to implement all three activities in the peer support meetings. One peer supporter put it this way:

*“I was prepared for meeting someone who had a goal, who had signed up because he/she wanted to make improvements, and X (the peer, red.), has never wanted that.“* (Peer supporter).

#### Encouragement and energy

Another group of interrelated mechanisms that generated both the intended cognitive and behavioural changes in DSM and use of healthcare services was the encouragement received from the peer supporter and the peer’s level of energy to interact in the intervention. Encouragement from peer supporters to regularly attend diabetes-related appointments with the GP or get started with healthier eating or exercise patterns positively affected the peers’ levels of energy to interact in the intervention and set meaningful, achievable goals. However, this was only seen in cases where peers’ individual contextual factors facilitated this engagement. In the following interview excerpt, the diabetes nurse described a case where encouragement from the peer supporter to improve eating habits and attend diabetes-related appointments with the GP was the push needed for the peer to make these behavioural changes:

*“Now, all of a sudden, there are some people around him who support him in the importance of visiting the GP (…). He knows what he has to do, but his challenge is in getting it done. (…) ‘Together on Diabetes’ has helped him get things done and that is what makes the big difference regarding his health, and health condition as I see it. It’s simply a matter of getting that little push.”* (Diabetes nurse).

In contrast, encouragement from the peer supporters did not generate outcomes among peers for whom contextual factors functioned as barriers. In the interviews, many described how their unstable financial situation, poor state of health condition, lack of energy and other life events resulted in them not being able to respond to their peer supporters’ encouragement. As one peer described:

*“I appreciate when somebody tries to help me, you know? Try to lift me (…) what you tell me now, I won’t do tomorrow but it is on my mind, and I try (…)”* (Peer, no outcomes achieved).

As this quote illustrates, the peer supporter’s encouragement activates a reflection within the peer. However, the peer’s challenging life circumstances and lack of energy constitute a barrier to respond and make the behavioural changes needed.

#### Experience of social and emotional support

The experience of receiving social and emotional support was found as a mechanism that generated increased self-care awareness (output). Unlike the other mechanisms, it was found within all peers regardless of their occupation and financial situation, health condition, amount of energy, and other life events. In the interviews, the peers emphasised how they valued the regular meetings with their peer supporter. In the majority of cases, these meetings were the only social contact they had. Many mentioned the importance of the peer supporter as a voluntary like-minded person that they could talk to about issues related to everyday life with T2D. One peer elaborated:

*“When I talk with a person, who is like me, who has diabetes, it is easier to explain because he understands (…) because he has the same problems as me.” (Peer, no outcomes achieved)*.

Several described being in the same situation as their peer supporter – in contrast to the feeling they experience with some healthcare professionals, where they sometimes feel judged:

*“When she has diabetes, and I have diabetes, we are kind of conspirators (…) Then the relationship becomes a little closer compared to if it was, for example, a doctor who probably always is set on keeping a distance (…) And I like that. That you are not judged all the time.” (Peer, outcomes achieved)*.

### ICAMO-models

Based on our empirical findings, we revised our initial ICAMO model. We developed two ICAMO models (Figs. 2 and 3), to illustrate how the identified contextual factors in peers’ everyday lives either facilitated or hindered their interactions in the intervention, thus affecting how the mechanisms within the peers were at stake. Figure 2 illustrates the ICAMO for the peers who achieved the intended outcomes and Fig. 3 illustrates the ICAMO for the peers who did not achieve these outcomes.

## Discussion

### Discussion of the findings

In this multi-method case study, we contribute with novel findings on the mechanisms that generated outcomes in a Danish peer support intervention for socially vulnerable people with T2D (peers). Furthermore, we provide in-depth insights into how individual contextual factors in peers’ everyday lives affected how the mechanisms were at stake. By categorising the peers, depending on whether these contextual factors facilitated or hindered the mechanisms to generate outcomes, we support Pawson & Tilley’s [25] notion that mechanisms only operate when the circumstances are right and that effective interventions depend on contextual awareness.

The study population in this case study represents the target group we would like to examine, as their sociodemographic characteristics are consistent with the results from the initial quantitative analysis conducted within the CCDC to define socially vulnerable people with T2D [22].

Compared to our initial ICAMO model, our analysis pinpoints two groups of mechanisms that improved the peers’ DSM and use of healthcare services (‘perceived needs and readiness’ and ‘encouragement and energy’). Independent of the influence from the contextual factors, a third mechanism, ‘experience of social and emotional support’, was identified within all peers to increase self-care awareness (output).

Although we present the mechanisms separately, we consider them interrelated. For example, we found that the peer supporters’ encouragement combined with the peers’ levels of energy only led to health behaviour changes in DSM if the peers had a perceived need and sufficient readiness to adopt and maintain healthier eating and exercise patterns. Furthermore, we found that the implementation of a minimum of two of the three intervention activities was needed to activate the outcome-generating mechanisms. As described by Pawson & Tilley, it is often impossible to find a key mechanism that is overarching and enables the outcome. Thus, the interaction of the intervention components, mechanisms and context as a whole facilitated the outcomes [25]. Furthermore, we found four contextual factors in peers’ everyday lives that either facilitated or hindered the mechanisms: ‘occupation and financial situation’, ‘health condition’, ‘energy’ and ‘other life events’. Not all contextual factors were present in all cases. As argued by Dalkin et al. [37] and Craig et al. [24], the distinction between context and mechanism can be difficult to make. We experienced this challenge, especially as both our mechanism and context focus were on the peers. For example, ‘energy’ arose both as a mechanism and contextual factor, as it was a product of a range of different individual contextual factors.

Relating our results to existing literature on peer support for people with T2D is challenging. Many peer support interventions contain other approaches (e.g., group- or telephone-based) targeted to other populations (not socially vulnerable). Moreover, they are delivered by other types of stakeholders (e.g., clinics, churches and community organisations) and providers (peer educators, community health workers and peer-partners) [9, 13, 44]. Furthermore, they are implemented in other national contexts with other welfare systems and thus differ markedly from the Danish context. For example, in the US, most peer support interventions are community-based and contain diabetes education [45]. Thus, they provide diabetes rehabilitation services similar to those that Danish municipalities are responsible for [46]. Finally, the existing studies on peer support have either focused on measuring the intervention effect (effect evaluation) [13] or fidelity issues in process evaluations [47]. Thus, our focus on the outcome-generating mechanisms and contextual factors influencing this process is novel to this field. However, there seem to be some recurrent characteristics that might be general for the implementation of peer support interventions. A scoping review on community-based peer-led health promotion programmes (not specific to T2D) reported similar findings to this study. For example, peers’ health conditions, work situations and beliefs towards the programme according to their needs were found as contextual factors to programme participation and engagement [48]. These contextual factors are also reported in the existing literature on DSM [15, 16, 49]. Likewise, lack of energy [16, 50] and the importance of social and emotional support have been addressed [50, 51].

### Strengths and limitations of the study

This study has several strengths. First, it builds on a realist evaluation approach to identify the mechanisms that generate the intended outcomes. Using the ICAMO framework [26] to structure our analytical findings, we demonstrate how each intervention activity activates different mechanisms within the peers. Furthermore, we analyse how they, in interaction with the specific context, generate different cognitive (outputs) and behavioural changes (outcomes). These are findings that would otherwise not be possible to discover through more classic RCT-studies. Second, the use of case studies, which was recommended in the RE approach when analysing complex interventions [43, 52], allowed us to test our initial ICAMO model and verify whether propositions in the ICAMO could be reproduced in different contexts. Third, it triangulates different data collection sources and both qualitative and quantitative methods, enhancing the credibility of the findings. Furthermore, with our relatively large sample size of nine cases with four different types of informants in each (*n* = 25), we achieved sufficient information power to test and revise our ICAMO models [53]. Likewise, were the peer supporters, the diabetes nurse, and the project manager able to describe some of the peers’ difficulties that the peers could not explain themselves.

However, the study has some limitations. First, due to the COVID-19 pandemic, seven interviews (three with peers) were conducted via telephone. Comparing these with the other interviews, we do not consider that this had consequences for the analysis. Second, we only investigated contextual factors in peers’ everyday lives. Another context focus (e.g., organisational or interpersonal contextual factors) [36] had provided us with other analytical findings. Third, we only used the ICAMO framework to structure our findings. Thus, we did not apply the realist interview. However, we did not consider this approach relevant, as we had in-depth knowledge from all key stakeholders to understand their perspectives. Furthermore, using the ICAMO framework contributes to the understanding of the interaction between context and mechanisms, and it may be relevant for other researchers to apply this when studying interventions targeting socially vulnerable groups. We believe that the in-depth data and analysis were valuable in generating contextually relevant evidence for improving the efficiency and effectiveness of peer support targeting socially vulnerable people with T2D and informing policy decisions for this group.

### Implication for practice and future research

The role of context in population health intervention research is increasingly acknowledged. In a guidance by Craig et al., the context can be taken into account by ensuring that the intervention is adaptable to the target group, for example, by conducting a needs assessment and testing the intervention in different contexts to uncover contextual factors that could affect the outcomes [24]. Therefore, we contribute to moving the field forward beyond having contextual awareness of the target population [23, 24]. The focus on the target group in the present study revealed that even though the Together on Diabetes intervention was developed to support socially vulnerable people with T2D, the most vulnerable people did not achieve the intended outcomes. Althoughthis group did not improve their DSM and/or increase their use of healthcare services, the peer supporters’ social and emotional support was still experienced as valuable and increased self-care awareness. We, therefore, find the social and emotional support important but not sufficient when it comes to a change in DSM and the use of healthcare services for this specific vulnerable group.

A key precondition for a successful programme is that the target groups’ social conditions are thoroughly addressed and a formal collaboration between health professionals and social workers is established. Also, it is a prerequisite for a successful programme, that each individual’s ’ most urgent social issues are taken care of when involving them in the patient education. Thus, the intervention required for this vulnerable group cannot be conducted solely by a group of voluntary peer supporters as the social conditions are much too multi-faceted and complicated.

## Conclusions

In this study, we explored the mechanisms generating outcomes in a Danish peer support intervention targeted to improve diabetes self-management and the use of healthcare services among socially vulnerable people with type 2-diabetes (peers). Using a realist evaluation approach, our study contributes novel, in-depth findings to a research field that needs more knowledge on how to reach socially vulnerable people with T2D with complex health interventions; people that the healthcare system, even in a universal welfare system such as in Denmark, does not reach. We found that the peer support intervention only activated mechanisms generating the intended outcomes if contextual factors in peers’ everyday lives facilitated their engagement in the intervention. Thus, we highlight the importance of having contextual awareness on the target group in designing and evaluating peer support interventions for socially vulnerable people with type 2-diabetes.

## Data Availability

The data/transcripts used during this study are available from the corresponding author upon reasonable request.

## Abbreviations

DSM: Diabetes self-management
T2D: Type 2-diabetes
RE: Realist evaluation
ICAMO: Intervention-context-actor-mechanism-outcome
CFD: Center for Diabetes
DDA: The Danish Diabetes Association
GP: General practitioner
CCDC: Cities Changing Diabetes Copenhagen (In Copenhagen the CCD partnership is between the Municipality of Copenhagen, the University of Copenhagen, Steno Diabetes Center Copenhagen, the Danish Diabetes Association and Novo Nordisk)
